# Pro-Cognitive and Antioxidant Effects of A-366, a Dual G9a Inhibitor and Histamine H3 Receptor Antagonist, in BTBR T+tf/J Mice

**DOI:** 10.1192/j.eurpsy.2026.10722

**Published:** 2026-07-31

**Authors:** M. Hajar, H. Stark, B. Sadek

**Affiliations:** 1 Pharmacology and Therapeutics, College of Medicine and Health Sciences, Al Ain, United Arab Emirates; 2 Institute of Pharmaceutical and Medicinal Chemistry, Heinrich Heine University Düsseldorf, Düsseldorf, Germany

## Abstract

**Introduction:**

Cognitive deficits represent a critical therapeutic challenge in neurodevelopmental and neuropsychiatric disorders. Epigenetic modulation and histaminergic neurotransmission are both recognized as promising targets for enhancing cognition. A-366 is a potent dual G9a inhibitor and histamine H3 receptor antagonist with potential pro-cognitive properties. This study evaluated the therapeutic effects of chronic A-366 treatment on learning, memory, and oxidative stress in BTBR T+tf/J mice.

**Objectives:**

This study aimed to evaluate the effects of chronic A-366 treatment on cognitive performance and oxidative stress in the BTBR T+tf/J mouse model.

**Methods:**

Male BTBR T+tf/J mice received chronic intraperitoneal administration of A-366 (0.5–2 mg/kg) for 21 days. Cognitive behaviors were assessed using the Fear Conditioning Test (FCT), Novel Object Recognition Test (NORT), and Open Field Test (OFT). Following behavioral evaluation, hippocampal tissues were analyzed for oxidative stress markers, including superoxide dismutase (SOD) activity and malondialdehyde (MDA) levels.

**Results:**

Chronic A-366 treatment significantly enhanced cognitive performance in BTBR mice, as demonstrated by improved contextual and cued memory in FCT, higher discrimination indices in NORT, and preserved exploratory activity in OFT. Biochemically, A-366 increased SOD activity and decreased MDA levels in hippocampal tissue, indicating attenuation of oxidative stress.

**Conclusions:**

A-366 exerts robust pro-cognitive effects in BTBR mice, potentially through dual modulation of epigenetic pathways and histaminergic neurotransmission, accompanied by restoration of oxidative balance. These findings highlight A-366 as a promising lead compound for the development of novel therapeutics targeting cognitive dysfunction in neurodevelopmental and neuropsychiatric disorders.

**Disclosure of Interest:**

None Declared

